# Dietary restriction of tyrosine and phenylalanine lowers tyrosinemia associated with nitisinone therapy of alkaptonuria

**DOI:** 10.1002/jimd.12172

**Published:** 2020-01-13

**Authors:** Juliette H. Hughes, Peter J. M. Wilson, Hazel Sutherland, Shirley Judd, Andrew T. Hughes, Anna M. Milan, Jonathan C. Jarvis, George Bou‐Gharios, Lakshminarayan R. Ranganath, James A. Gallagher

**Affiliations:** ^1^ Department of Musculoskeletal Biology I, Institute of Ageing and Chronic Disease University of Liverpool Liverpool UK; ^2^ Department of Nutrition and Dietetics Royal Liverpool University Hospital Trust Liverpool UK; ^3^ Liverpool Clinical Laboratories, Department of Clinical Biochemistry and Metabolic Medicine Royal Liverpool and Broadgreen University Hospitals Trust Liverpool UK; ^4^ School of Sport and Exercise Sciences, Faculty of Science Liverpool John Moores University Liverpool UK

**Keywords:** alkaptonuria, nitisinone, phenylalanine, protein, tyrosinemia, tyrosine

## Abstract

Alkaptonuria (AKU) is caused by homogentisate 1,2‐dioxygenase deficiency that leads to homogentisic acid (HGA) accumulation, ochronosis and severe osteoarthropathy. Recently, nitisinone treatment, which blocks HGA formation, has been effective in AKU patients. However, a consequence of nitisinone is elevated tyrosine that can cause keratopathy. The effect of tyrosine and phenylalanine dietary restriction was investigated in nitisinone‐treated AKU mice, and in an observational study of dietary intervention in AKU patients. Nitisinone‐treated AKU mice were fed tyrosine/phenylalanine‐free and phenylalanine‐free diets with phenylalanine supplementation in drinking water. Tyrosine metabolites were measured pre‐nitisinone, post‐nitisinone, and after dietary restriction. Subsequently an observational study was undertaken in 10 patients attending the National Alkaptonuria Centre (NAC), with tyrosine >700 μmol/L who had been advised to restrict dietary protein intake and where necessary, to use tyrosine/phenylalanine‐free amino acid supplements. Elevated tyrosine (813 μmol/L) was significantly reduced in nitisinone‐treated AKU mice fed a tyrosine/phenylalanine‐free diet in a dose responsive manner. At 3 days of restriction, tyrosine was 389.3, 274.8, and 144.3 μmol/L with decreasing phenylalanine doses. In contrast, tyrosine was not effectively reduced in mice by a phenylalanine‐free diet; at 3 days tyrosine was 757.3, 530.2, and 656.2 μmol/L, with no dose response to phenylalanine supplementation. In NAC patients, tyrosine was significantly reduced (*P* = .002) when restricting dietary protein alone, and when combined with tyrosine/phenylalanine‐free amino acid supplementation; 4 out of 10 patients achieved tyrosine <700 μmol/L. Tyrosine/phenylalanine dietary restriction significantly reduced nitisinone‐induced tyrosinemia in mice, with phenylalanine restriction alone proving ineffective. Similarly, protein restriction significantly reduced circulating tyrosine in AKU patients.

## 1 INTRODUCTION

**Figure 1:**
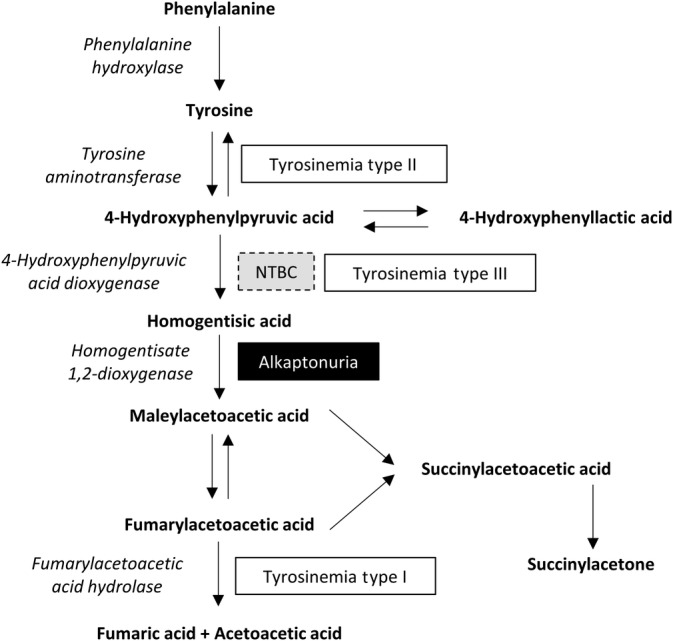
Tyrosine metabolism pathway. Enzymes are in italics. Disorders are in boxes. NTBC (nitisinone) inhibits 4‐hydroxyphenylpyruvic acid dioxygenase

Alkaptonuria (AKU; OMIM #203500) is an autosomal recessive, multisystem disease caused by mutations in the homogentisate 1,2‐dioxygenase (HGD) enzyme (EC 1.13.11.5).^1^ HGD deficiency leads to homogentisic acid (HGA) accumulation (Figure 1A) in the blood and tissues. Over time, excess HGA forms a dark brown ochronotic pigment that deposits in connective tissues causing them to become stiff and brittle,^2^, ^3^, ^4^, ^5^ leading to early‐onset and severe osteoarthropathy.

Recently, a drug called nitisinone (2‐(2‐nitro‐4‐[trifluoromethyl]benzoyl)cyclohexane‐1,3‐dione; NTBC) that blocks 4‐hydroxyphenylpyruvic acid dioxygenase (HPPD; EC 1.13.11.27) which converts 4‐hydroxyphenylpyruvic acid (HPPA) into HGA was shown to prevent ochronosis in AKU mice,^6^, ^7^ leading to a series of human clinical trials assessing nitisinone in AKU.^8^ The SONIA‐1 trial concluded that nitisinone effectively reduces HGA to a level likely to prevent ochronosis.^8^ However, increased serum tyrosine was seen with nitisinone treatment.^8^

Elevated serum tyrosine (tyrosinemia) causes eye and skin keratopathy in nitisinone‐treated hereditary tyrosinemia type 1 (HT‐1; OMIM #276700),^9^ resembling the dermal and ocular symptoms seen in hereditary tyrosinemia type II (OMIM #276600).^10^ Low‐dose nitisinone (0.5 mg daily) in an AKU patient has induced ocular irritation and photophobia that presented before corneal crystal deposition.^11^ More worryingly, a study reported mild corneal keratopathy in an AKU patient on 2 mg of nitisinone daily with no ocular symptoms or pain.^12^ This is concerning as untreated keratopathy could be sight‐threatening if not detected.

Moreover, the association of tyrosinemia with neural cognition is another important consideration, as tyrosine is a precursor to neurotransmitters such as dopamine, adrenaline and noradrenaline. Metabolites of these neurotransmitters have previously been shown to significantly alter with nitisinone treatment in AKU patients.^13^ Similarly, numerous reports and studies document impaired cognitive function in HT‐1 patients that have been treated long‐term with nitisinone and protein restriction.^9^, ^14^, ^15^ These neurodevelopmental effects however could be due to either nitisinone‐induced tyrosinemia or severe liver failure experienced before diagnosis and subsequent initiation of nitisinone treatment. With the cause of impaired cognition in HT‐1 remaining to be elucidated, the off‐license use of nitisinone has been restricted to ≥16 years in AKU.

A tyrosine‐lowering strategy that can prevent keratopathy, such as protein/tyrosine restriction, is implemented in conjunction with nitisinone treatment in HT‐1.^16^, ^17^ However, poor adherence to protein supplements coupled with an inadequate low protein diet, can lead to essential amino acid and micronutrient deficiency.^18^, ^19^ Although advised, no clinical study has investigated the effectiveness of dietary restriction at reducing nitisinone‐induced tyrosinemia in AKU.

Here, dietary restriction of tyrosine and phenylalanine was investigated in nitisinone‐treated AKU mice to establish its effectiveness at reducing tyrosinemia. Additional observational data of dietary intervention in AKU patients attending the National Alkaptonuria Centre (NAC; Liverpool, UK), where patients receive 2 mg nitisinone daily, are presented. At the NAC, serum tyrosine increases to a mean level of approximately 600 to 700 μmol/L^20^, ^21^ with nitisinone, therefore patients are advised to restrict protein intake with guidance from a specialist dietician. We provide proof‐of‐concept that mechanisms reducing uptake of dietary tyrosine into the bloodstream would be effective.

## 2 METHODS

### 2.1 Mice

BALB/c Hgd−/− mice,^7^ referred to as AKU, were all housed and maintained within the University of Liverpool Biological Services Unit in specific pathogen‐free conditions in accordance with Home Office UK guidelines. Food and water were available ad libitum.

### 2.2 Dietary restriction studies

To investigate tyrosine and phenylalanine dietary restriction on nitisinone‐induced tyrosinemia and on other tyrosine pathway metabolites, AKU mice (n = 6; three male, three female) were treated with nitisinone for 1 week on normal diet, then switched to either tyrosine/phenylalanine‐free or phenylalanine only‐free diets with phenylalanine supplemented into the drinking water to investigate dose response, while still on nitisinone. Controls remained on normal diet. Blood samples were taken pre‐nitisinone, 1 week post‐nitisinone and then during dietary restriction.

### 2.3 Diets

The normal control mouse diet (CRM(P), product code 801722, Special Diet Services [SDS], UK), contained 0.88% phenylalanine and 0.59% tyrosine. Restricted diets were synthetically made by SDS in pellet form. Synthetic raw materials were used to produce an amino acid defined diet which has an amino acid profile similar to the normal CRM(P) diet, with tyrosine and/or phenylalanine removed and balanced on maize starch.

### 2.4 Nitisinone and phenylalanine provision in drinking water

Nitisinone was added to the drinking water at 4 mg/L (0.8 mg/kg; based on a 30 g mouse drinking approximately 6 mL/day) and was freely available. Phenylalanine was added to the drinking water at various doses, between 0 and 5 mg/mL as indicated in the results.

### 2.5 Calculating dietary restriction

Daily food and water intake is approximately 5 g and 6 mL respectively, per 30 g bodyweight for the BALB/cByJ strain.^22^ A 30 g mouse on the normal CRM(P) diet therefore consumes approximately 44 mg/day phenylalanine and 29.5 mg/day tyrosine. In the tyrosine/phenylalanine‐free diet study, drinking water was supplemented with 5, 2.5, and 1.25 mg/mL phenylalanine, equating to approximately 68% phenylalanine/0% tyrosine, 34% phenylalanine/0% tyrosine and 17% phenylalanine/0% tyrosine, respectively. In the phenylalanine free diet study, drinking water was supplemented with 2.5, 0.625, and 0 mg/mL phenylalanine, equating to approximately 34% phenylalanine/100% tyrosine, 8.5% phenylalanine/100% tyrosine and 0% phenylalanine/100% tyrosine, respectively.

### 2.6 Blood collection from mice

Acidified plasma from venous tail bleeds was collected to analyze tyrosine pathway metabolites via HPLC tandem mass spectrometry.^23^

### 2.7 NAC patients

NAC patients receive off‐license treatment of 2 mg nitisinone daily, dietary advice and have serum tyrosine monitored at yearly visits.^21^ The NAC aims to achieve tyrosine <500 μmol/L. Tyrosine thresholds of 500 to 700 μmol/L and 700 to 900 μmol/L are used as guidelines for reducing dietary protein consumption to 0.9 and 0.8 g protein/kg bodyweight/day, respectively. Patients with tyrosine >700 μmol/L are more closely monitored with additional blood sampling if required. Fasting blood samples were taken in the morning. Ten anonymized NAC patients with tyrosine >700 μmol/L are reported here. All patients received advice to reduce dietary protein intake to a recommended minimum level of 0.75 g protein/kg bodyweight/day, to reduce circulating tyrosine while meeting minimum nutritional requirements. A TYR cooler (Vitaflo, UK) protein substitute (free in tyrosine/phenylalanine) drink was used by seven of 10 patients. TYR cooler dosage was determined by the dietician for each individual; protein removed from the diet, below the recommended minimum, was exchanged for the equivalent amount of protein in the cooler. These patients had been attending the NAC for a mean of 36.5 months (range 29‐48) at the initial tyrosine concentration, with a mean age of 56 years (range 29‐71). The gender of patients was five male, five female. Eight were white British, one was Indian, and one was Pakistani.

### 2.8 Statistical analysis

Statistical analysis was performed using Stats Direct 3 statistical software (England, UK). Means are represented as mean ± SE on the mean (SEM). Statistical significance, using *α* = 0.05; *P < .05; **P < .01; ***P < .001. Error bars represent SEM.

## 3 RESULTS

### 3.1 Effect of nitisinone treatment on tyrosine pathway metabolites

**Figure 2:**
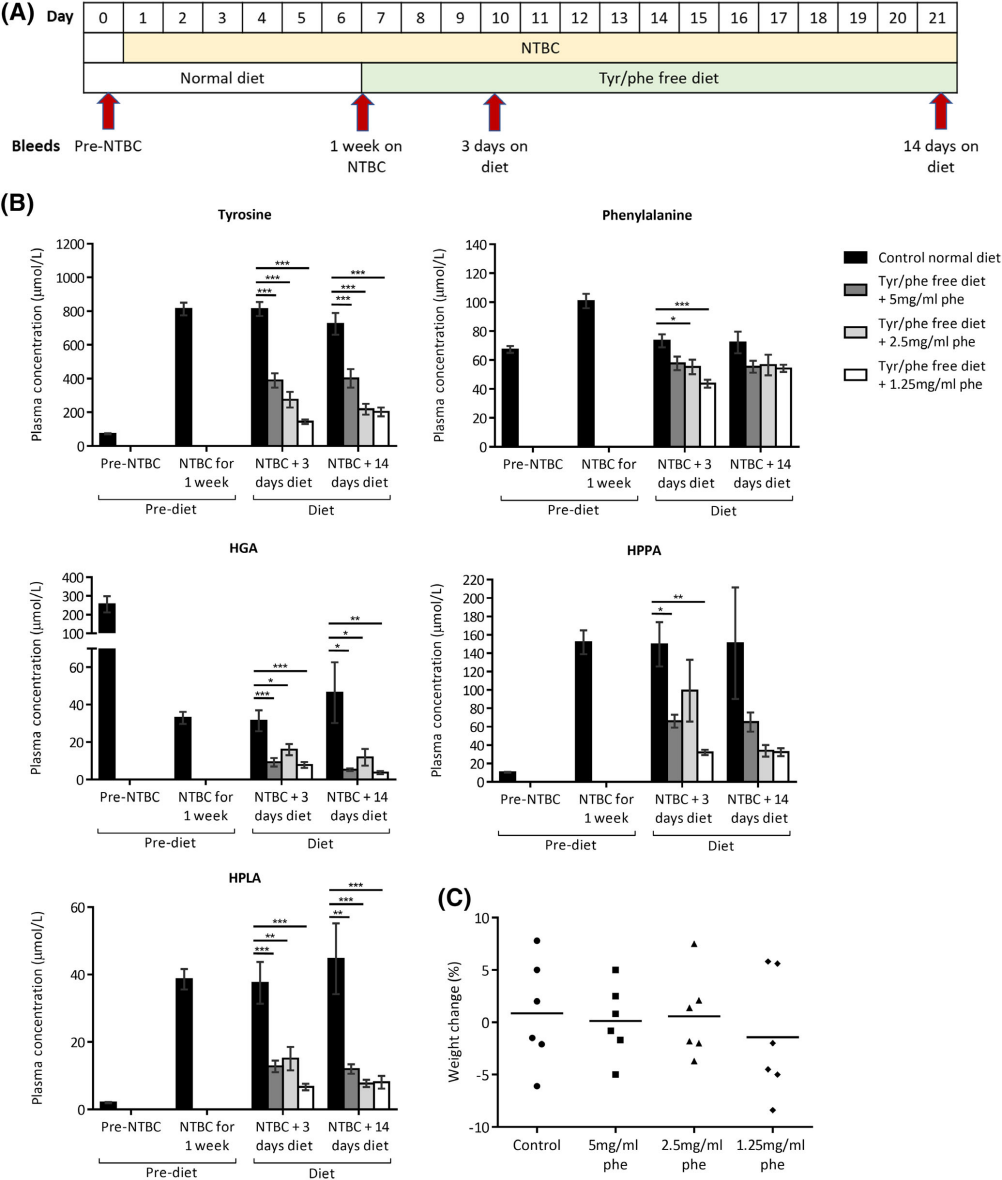
Dietary restriction of tyrosine and phenylalanine in NTBC‐treated AKU mice. A, shows when blood samples were taken and when diet conditions were altered. B, shows plasma metabolite levels before NTBC treatment (all mice; n = 24), after 1 week of NTBC treatment (all mice; n = 24), then after 3 (n = 6) and 14 days (n = 6) of tyrosine/phenylalanine restriction. C, shows the change in bodyweight of individual mice in each group from day 7 (start of dietary restriction) and day 21 (after 14 days of dietary restriction). HGA, homogentisic acid; HPLA, 4‐hydroxyphenyllactic acid; HPPA, 4‐hydroxyphenylpyruvic acid; NTBC, nitisinone. Error bars represent SEM. One‐way ANOVA (Tukey post‐hoc) significance: *P < .05; **P < .01; ***P < .001

Tyrosine pathway metabolites were measured in the plasma of 24 AKU mice pre‐ and post‐nitisinone treatment (4 mg/L in drinking water for 7 days) (Figure 2B). HGA was significantly decreased 8‐fold from 255.1 ± 43.2 to 32.9 ± 3.2 μmol/L while all other metabolites increased; all changes were significant (*P* < .001, two‐tailed paired *t* test). Tyrosine increased 11‐fold from 72.9 ± 3.6 μmol/L to 813.3 ± 37.6 μmol/L. Phenylalanine increased 1.5‐fold from 67.3 ± 2.4 to 100.8 ± 4.9 μmol/L. HPPA increased 14.5‐fold from 10.5 ± 0.4 to 151.8 ± 12.9 μmol/L. 4‐hydroxyphenyllactic acid (HPLA) increased 19‐fold from 2.0 ± 0.2 to 38.6 ± 3.0 μmol/L.

### 3.2 Tyrosine/phenylalanine dietary restriction

Nitisinone‐treated AKU mice were fed a tyrosine and phenylalanine‐free diet, with phenylalanine supplemented into the drinking water (5‐1.25 mg/mL). The control group remained on a normal diet. Blood samples were taken according to the scheme in Figure 2A. Three days after dietary restriction, tyrosine was reduced from 813.3 ± 37.6 μmol/L (all groups, n = 24; Figure 2B) in a dose responsive manner to 389.3 ± 42.3, 274.8 ± 6.3, and 144.3 ± 13.0 μmol/L in the 5, 2.5, and 1.25 mg restricted groups, respectively. After 14 days of restriction, these reductions were maintained. All comparisons to the control group at both 3 and 14 days were significant (*P* < .001).

After 3 days of dietary restriction, significant reductions in phenylalanine, HGA, HPPA and HPLA were present, however the dose response was not as clear in these metabolites (Figure 2B). After 14 days of dietary restriction, only HGA and HPLA showed significant reductions compared to the control group.

A second tyrosine/phenylalanine dietary restriction study (data not shown) was carried out, with lower doses of phenylalanine supplementation in the water (0.625, 0.3125, and 0 mg/mL). Further restriction of phenylalanine however did not result in lower plasma tyrosine concentrations than those shown in Figure 2, at both 3 and 4 days of restriction. No significant weight changes were seen between any of the groups at 4 days of restriction.

### 3.3 Phenylalanine only dietary restriction

**Figure 3:**
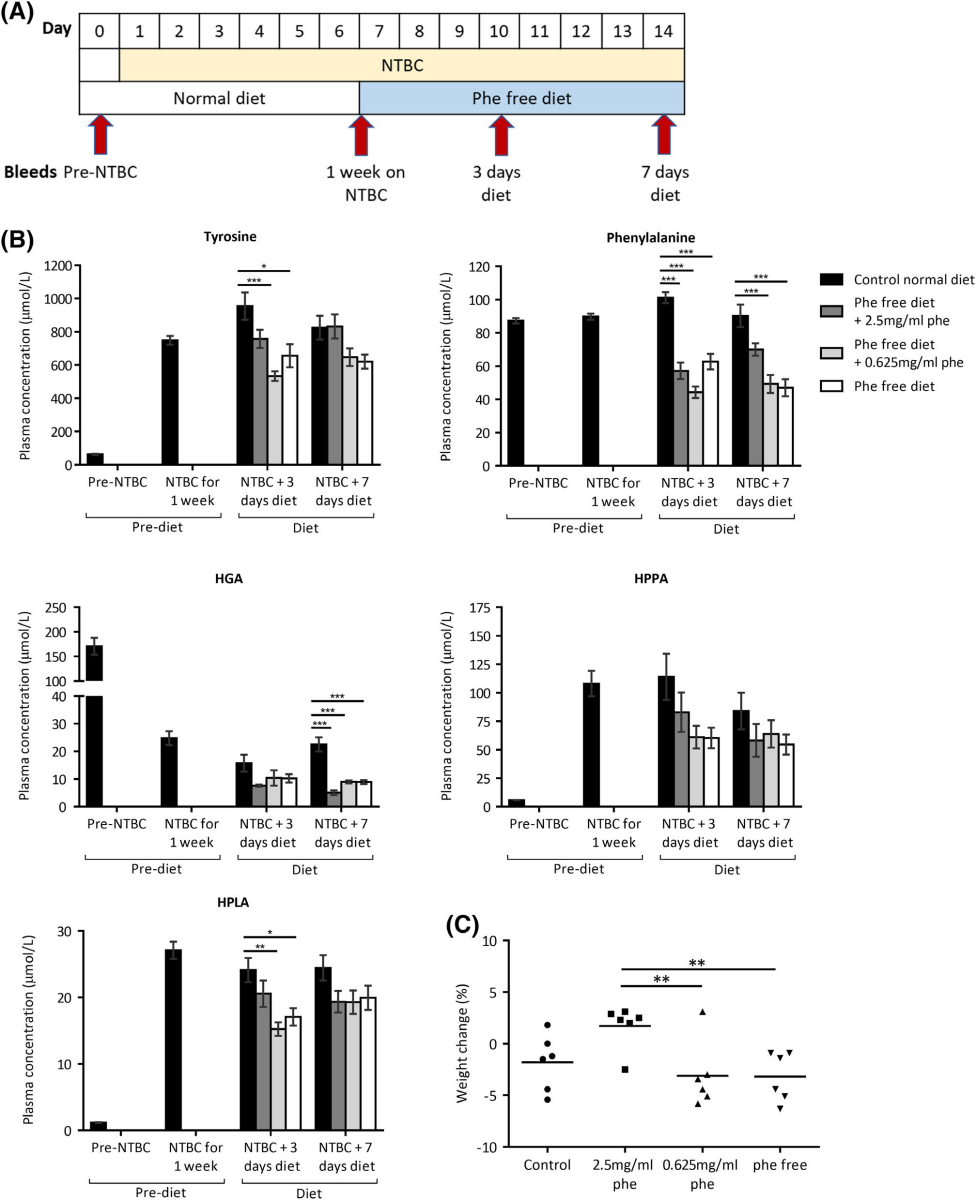
Dietary restriction of phenylalanine in NTBC‐treated AKU mice. A, shows when blood samples were taken and when diet conditions were altered. B, shows the plasma metabolite levels before NTBC treatment (all mice; n = 24), after 1 week of NTBC treatment (all mice; n = 24), then after 3 (n = 6) and 14 (n = 6) days of diet phenylalanine restriction. C, shows the change in bodyweight of individual mice in each group from day 7 (start of dietary restriction) and day 14 (after 7 days of dietary restriction). HGA, homogentisic acid; HPLA, 4‐hydroxyphenyllactic acid; HPPA, 4‐hydroxyphenylpyruvic acid; NTBC, nitisinone. Error bars represent SEM. One‐way ANOVA (Tukey post‐hoc) significance: *P < .05, **P < .01, ***P < .001

Nitisinone‐treated AKU mice were fed a phenylalanine‐free diet with normal tyrosine, with phenylalanine supplemented in drinking water (2.5‐0 mg/mL). The control group remained on a normal diet. Blood samples were taken according to the scheme in Figure 3A. Plasma tyrosine was 63.5 ± 2.7 μmol/L pre‐nitisinone and increased to 748.9 ± 26.8 μmol/L post‐nitisinone (n = 24, all groups, Figure 3B). After 3 days of phenylalanine restriction, tyrosine levels were 757.3 ± 54.9, 533.2 ± 28.8, and 656.2 ± 69.6 μmol/L, with only the latter two groups, (0.625 mg/mL and phenylalanine‐free), being significantly different to control (*P* < .001 and *P* = .014, respectively). After 7 days of dietary restriction, none of the restricted groups had significantly lower tyrosine compared with the control group.

Significant reductions in plasma phenylalanine were seen at both 3 days (all groups *P* < .001 compared with control) and after 7 days (0.625 mg/mL and phenylalanine‐free, both *P* < .001 compared with control; 2.5 mg/mL not significantly different, *P* = .065) of phenylalanine dietary restriction. HPLA demonstrated significant differences after 3 days in the 0.625 mg/mL and phenylalanine‐free groups however these significant differences were not maintained after 7 days. HGA showed significant differences after 7 days of dietary restriction in all the restricted groups however these differences were not seen after 3 days. HPPA was not significantly reduced in the restricted groups compared with control, at neither 3 nor 7 days of dietary restriction.

### 3.4 Body weight

The extent of tyrosine/phenylalanine dietary restriction (%) in these studies was based upon daily food and water intake values for mice weighing 30 g. At day 7 when dietary restriction was implemented, the mean body weight of all mice (n = 24) was 29.8 g (23.5‐35.7 g) and 30.0 g (23.7‐37.1 g) in the tyrosine/phenylalanine‐free and phenylalanine only‐free diet studies respectively.

Individual body weight was monitored over the duration of dietary restriction. At 14 days of restriction in the tyrosine/phenylalanine‐free diet study, the restricted groups showed no significant change in body weight (Figure 2C) compared to the control group (*P* = .841).

Figure 3C shows the changes in body weight after 7 days of restriction in the phenylalanine only diet study. One‐way ANOVA demonstrated a significant difference in weight change between the groups (*P* = .014), with differences found between the 2.5 mg/mL group compared with the 0.625 mg/mL (*P* = .023) and phenylalanine‐free (*P* = .021) groups (post‐hoc Tukey test). None of the restricted groups however showed weight changes significantly different to the control group.

### 3.5 NAC dietary intervention in AKU patients

Average estimated protein intake from 7‐day food diaries in all AKU patients attending the NAC was 1.1 g protein/kg bodyweight/day at baseline visit 1 (unpublished data) before any intervention, which is comparable with the general population (NDNS2016). With nitisinone, dietary protein intake is restricted to a recommended minimum level of 0.75 g protein/kg bodyweight/day (SACN/COMA2008/2017), with additional metabolic requirements provided by prescribed tyrosine/phenylalanine‐free amino acid supplements in some patients.

**Figure 4:**
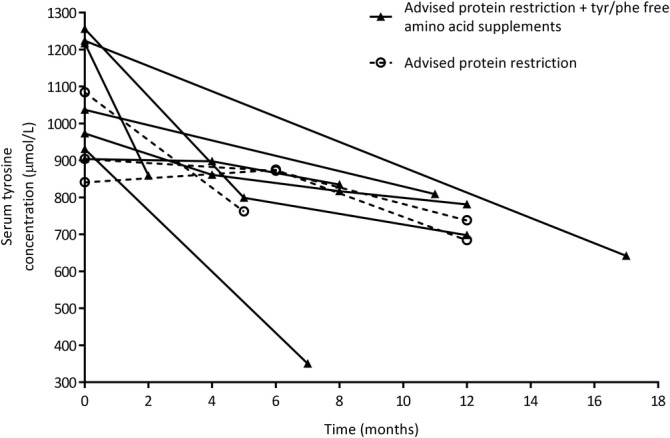
Serum tyrosine from 10 AKU patients receiving nitisinone attending the National Alkaptonuria Centre. All 10 patients had tyrosine >700 μmol/L. Both dietary advice (restriction of dietary protein to a recommended minimum level of 0.75 g protein/kg bodyweight) (n = 3) and dietary advice in combination with tyrosine/phenylalanine‐free amino acid supplements (n = 7) reduced serum tyrosine. Initial tyrosine concentrations in the group using amino acid supplements were measured after dietary advice alone, with subsequent tyrosine concentrations measured after a combination of dietary advice and amino acid supplement use

Overall, significant reductions in serum tyrosine (Figure 4) were seen in the 10 NAC patients observed (*P* = .002; two‐tailed Wilcoxon's signed rank test), with four patients reducing serum tyrosine <700 μmol/L. Three patients (Figure 4, dashed lines) reduced serum tyrosine (mean[range]) by 22 [18‐30]% by advised protein restriction alone; the other seven patients required a combination of reduced protein intake with tyrosine/phenylalanine‐free supplements to achieve a 33 [8‐62]% reduction. The initial tyrosine value for patients using amino acid supplements was with dietary restriction alone, with subsequent values using a combination of reduced protein intake and amino acid supplementation. Across all 10 patients and all sample time points, phenylalanine (mean[range]) was 57 [38‐108] μmol/L.

## 4 DISCUSSION

The treatment of AKU with the HGA‐lowering drug nitisinone has proven to be effective at reducing HGA.^24^ However, data from clinical trial^8^ and off‐label prescription use at the NAC^21^ clearly demonstrate concomitant elevation of tyrosine. Tyrosinemia can cause eye and skin keratopathy in a minority of patients requiring a lower dose or cessation of nitisinone, or a strict low protein diet, to resolve symptoms. Low‐dose nitisinone, ranging from 0.5 to 2 mg daily, has caused eye complications in five reported AKU patients to date.^11^, ^12^, ^25^, ^26^ Attempting to restrict dietary tyrosine and phenylalanine consumption, while taking nitisinone to keep HGA low, is the most logical approach to combat tyrosinemia. A protein restricted diet however proves very difficult to maintain and often causes keratopathy to return when not adhered to.^12^ Therefore, finding an alternative strategy to reduce dietary uptake of tyrosine and/or phenylalanine would allow nitisinone‐treated AKU patients to consume a normal protein diet. Before such strategies are investigated, the findings of this paper has showed that reducing dietary tyrosine/phenylalanine is effective at reducing tyrosine in AKU mice, with similar observational evidence that lowering tyrosine via dietary intervention in patients is achievable, therefore establishing a proof of principle for targeting dietary tyrosine/phenylalanine in patients who are on nitisinone.

Tyrosine threshold recommendations with nitisinone treatment, which are often arbitrary, vary in the literature. In HT‐1 children <12 years, the recommended tyrosine level is 200 to 400 μmol/L, with concentrations after this age allowed to rise, with eye complications experienced >800 μmol/L.^16^ Another HT‐1 review of 22 centers treating patients aged 0 to 24 years found maximum acceptable tyrosine levels to be 200 to 800 μmol/L, with 400 μmol/L determined to be a safe and feasible target.^17^ A report of an adult AKU patient who experienced eye keratopathy while receiving low‐dose nitisinone (0.5‐1.5 mg daily) suggests a target tyrosine of <600 μmol/L,^11^ while a more recent NAC publication suggests <500 μmol/L is acceptable.^24^ NAC tyrosine thresholds are designed to fit a dietetic care plan, that aims to reduce serum tyrosine in order to prevent symptomatic keratopathy, with systematic removal of dietary protein, while taking into account compliance difficulties experienced in patients.

In this study, the mouse tyrosine/phenylalanine‐free diet clearly showed a dose‐responsive reduction in plasma tyrosine, with all groups reaching <400 μmol/L. The least restricted group (5 mg/mL) reduced tyrosine to 389 μmol/L at 3 days, achieved with approximately 68% phenylalanine/no tyrosine. We also showed evidence that human patients on nitisinone can reduce serum tyrosine with dietary intervention. Tyrosine was significantly lowered either by a low protein diet alone or in combination with prescribed tyrosine/phenylalanine‐free amino acid supplementation. Although four of 10 patients achieved tyrosine <700 μmol/L, tyrosine was greatly reduced in some patients, notably from 1084 to 762 μmol/L, and from 1217 to 859 μmol/L. Of the four patients that reduced tyrosine below 700 μmol/L, only one patient achieved this with dietary advice alone; the other three patients used amino acid supplementation. Prior to nitisinone, most adult AKU patients will have consumed a normal protein diet throughout their life, therefore adaption to dietary changes can be difficult, especially in the absence of tyrosine‐related keratopathy where no benefit is perceived.

The NAC patient data were not collected as a controlled trial, but as an observational study where patients are only advised to restrict their protein intake, therefore strict monitoring of compliance and protein intake was not carried out. After specialist advice, patients were responsible for restricting dietary protein in their daily lives, with guidance from the dietician if needed. Progress with dietary compliance is a long‐term iterative process, since achieving long‐term, sustained behavior change in adults is a recognized national dilemma. Compliance is the most likely reason that only one of 10 NAC patients achieved <500 μmol/L tyrosine and is why mice were used for the restriction experiments reported here. The diets of mice are easy to control, in addition to eliminating other confounding factors such as lifestyle, exercise, mobility, and disease severity differences. The mouse data provides evidence that dietary restriction of tyrosine/phenylalanine can effectively reduce nitisinone‐induced tyrosinemia, providing the rationale to carry out a controlled trial in human AKU patients, where lower tyrosine levels than those presented here would be expected.

In mice, the dose response observed due to phenylalanine supplementation, in the absence of tyrosine (Figure 2), suggests that tyrosine derived from phenylalanine is important to target. However, phenylalanine only restriction was ineffective at lowering nitisinone‐induced tyrosinemia, even with complete phenylalanine restriction (Figure 3). This is an important consideration as emerging experimental and dietary phenylalanine‐lowering strategies for the metabolic disease phenylketonuria (OMIM #261600) may have been beneficial for nitisinone‐induced tyrosinemia if phenylalanine restriction alone had proven effective. Such strategies include enzymatic phenylalanine degradation by exogenous phenylalanine ammonia lyase enzyme, diets supplemented with naturally occurring glycomacropeptide (GMP) protein that is naturally low in phenylalanine instead of synthetic amino acid foods, and genetically modified probiotics that target phenylalanine‐degradation in the intestine.^27^, ^28^

Due to phenylalanine being an essential amino acid, its restriction alone could lead to inadequate protein turnover, with the balance of catabolism/anabolism disrupted, causing a loss of lean mass.^29^ Although not significantly different to the control group in the phenylalanine only restriction study, there was a significant body weight reduction in the phenylalanine‐free and 0.625 mg/mL groups compared to the 2.5 mg/mL group (Figure 3) at 7 days. This weight loss was seen in the presence of tyrosine, suggesting that phenylalanine restriction could lead to catabolism, therefore targeting tyrosine would be more desirable. At 14 days of restriction, no significant weight changes were seen in Figure 2 in which tyrosine was absent and phenylalanine was partially present.

It is essential to balance lowering protein intake in order to lower tyrosine, with maintaining a healthy weight and muscle mass, since catabolism itself provides residual tyrosine. During routine NAC visits, anthropometric data such as grip, mid upper arm circumference, and bioimpedance data such as lean mass and fat mass, is collected to provide sequential evidence of body composition trends to minimize risk of catabolism. These parameters were not investigated in the AKU patients presented in Figure 4, as less than half of the tyrosine concentrations were measured at a routine (yearly) NAC visit, where such anthropometric data is collected. These parameters are worthy of investigation in both mice and humans in the future.

In addition to decreased tyrosine, other metabolic improvements were observed with dietary restriction in mice. HGA, which is lowered with nitisinone treatment, was then significantly lowered in the restricted groups compared to the control in the tyrosine/phenylalanine‐free study. Both HPPA and HPLA in mice, which were elevated with nitisinone as recently shown in human AKU patients,^30^ were significantly reduced compared to the control with restriction. Although the effect of elevated HPPA and HPLA is unknown, reducing them back toward normal physiological levels would be desirable. Phenylalanine in mice was significantly reduced after 3 days in the tyrosine/phenylalanine restriction study, but this was not maintained to 14 days. In human NAC patients, phenylalanine levels were within the normal reference range.

Nitisinone is a lifelong treatment for AKU and is associated with hypertyrosinemia. Removal of tyrosine/phenylalanine, but not phenylalanine alone, from the diet of mice is effective at reducing nitisinone‐induced tyrosinemia. Protein restriction coupled with tyrosine/phenylalanine‐free amino acid supplementation was able to reduce tyrosine levels in patients observed at the NAC, although these reductions were greater in mice, most likely owing to patient compliance. A controlled trial in AKU patients would likely see results similar to the mouse data shown here. We suggest that strategies reducing tyrosine uptake from the intestine, such as degradation of tyrosine by an exogenous enzyme or a tyrosine‐specific binder that prevents uptake into the circulation, that allow a normal protein diet to be consumed, could be investigated not only for nitisinone‐treated AKU patients, but also for nitisinone‐treated HT‐1 patients.

## AUTHOR CONTRIBUTIONS

J.H.H., P.J.M.W., H.S., S.J., A.T.H., and A.M.M. collected and analyzed data. Mouse data were collected under the project licence of J.C.J. and G.B.G. L.R.R. and J.A.G. were involved with study concept design and supervised the work. J.H.H. wrote the paper, with contributions from all authors. All authors approved the final version.

## ETHICS STATEMENT

The data collected from the NAC was approved by the Institutional Audit Committee (Audit No:ACO3836). All institutional and national guidelines for the care and use of laboratory animals were followed.
